# Association Between Academic, Initial Licensure, Employment Factors, and NCLEX-RN Performance of Philippine-Educated Nurses

**DOI:** 10.3390/ijerph22040653

**Published:** 2025-04-21

**Authors:** James Montegrico, Zhuo Job Chen

**Affiliations:** School of Nursing, University of North Carolina at Charlotte, Charlotte, NC 28223, USA; job.chen@charlotte.edu

**Keywords:** internationally educated nurses, NCLEX, nursing education, nursing workforce, Philippines

## Abstract

The United States’ nursing shortage attracted internationally educated nurses (IENs) to take the National Council Licensure Examination–Registered Nurses (NCLEX-RN), which is required to practice nursing in the U.S. Philippine-educated nurses (PENs) comprised more than half of IENs in the U.S. nursing workforce. From 2002 to 2021, only 45.8% of 177,730 PENs passed the exam. Published studies investigating IEN NCLEX-RN performance are limited. This study addresses this gap in the literature. This study determined the association between academic, initial nursing licensure, and employment factors on PEN NCLEX-RN pass rates. A retrospective correlation research design was used to determine the association among the research variables. Participants were recruited through online nursing groups. Descriptive statistics compared characteristics of PENs who passed or failed the NCLEX on the first attempt. Chi-squared and Fisher’s exact tests were used to determine the association between the research variables. Initial nursing licensure and nursing workplace were significantly associated with PENs passing the NCLEX-RN. Identifying unique PENs’ contextual characteristics is critical in preparing them to pass the NCLEX-RN. Findings provide input to educational and regulatory bodies to improve the NCLEX-RN individual outcomes and Philippine NCLEX-RN pass rates.

## 1. Introduction

Almost 20 percent of the world’s nursing workforce is in the United States (U.S.) [1]. The U.S. is the largest importer of internationally educated nurses (IENs), attracting nurses from 187 countries [2]. Of the 4.3 million U.S.-registered nurses (RN), 6–8% are IENs and more than half are Philippine-educated nurses (PEN) [3,4,5]. The cyclical U.S. nursing shortage attracted IENs to migrate to the U.S., as IEN recruitment became a common strategy to help address the nursing shortage. In March 2021, the Healthcare Workforce Resilience Act (S. 1024) was introduced in the U.S. Senate to allow 25,000 IENs to support the U.S. nursing workforce during the pandemic [6].

The National Council Licensure Examination–Registered Nurses (NCLEX-RN) is a requirement to practice nursing in the U.S. and Canada. From 2002 to 2021, out of 285,094 IEN first-time NCLEX-RN candidates, more than half failed (52.7%, *n* = 150,232), and only 45.8% (*n* = 81,393) of 177,730 PENs passed the NCLEX-RN at first attempt [2,7]. Furthermore, almost 75% of 68,949 IENs who repeated the NCLEX-RN from 2017 to 2021 failed [2].

The low first-time pass rate (FTPR) and limited literature on PEN NCLEX-RN performance are the main rationales for conducting this study. This study analyzed the association of academic, initial nursing licensure, and employment factors, and PEN NCLEX-RN performance to enhance understanding of this area that has significant implications for nursing education and workforce. Specifically, the research questions in this study are as follows: (1) What are the characteristics of PENs who passed or failed the NCLEX-RN at the first attempt? (2) What academic, initial licensure, and employment factors are associated with PEN NCLEX-RN performance? Most NCLEX-RN studies were conducted on U.S.-educated nurses (USEN) [8,9]. With limited research on PEN NCLEX-RN performance, this study addresses this gap in the literature. This study can guide prospective PENs in increasing their likelihood of passing the NCLEX-RN and has the potential to increase the pipeline of PENs in the U.S. Furthermore, the findings may provide input to nursing education in the Philippines, which is the primary IEN source country to the U.S. [2].

### 1.1. Conceptual Framework

Factors affecting NCLEX-RN performance are multi-dimensional. Jeffrey’s Nursing Universal Retention and Success (NURS) model was used as the conceptual framework to understand the multidimensionality of factors affecting nursing students’ success [10]. The NURS model postulates that nursing success, defined as positive academic and psychological outcomes, passing the NCLEX-RN, and employment, is an interplay of student profile characteristics such as age, language, and work experience; affective factors of cultural values, self-efficacy, and motivation; academic factors such as study habits and attendance; professional integration factors including faculty and enrichment programs; environmental factors like finances, family responsibilities, and employment; and outside surrounding factors including politics, economics, and health care system [10]. These factors were used in this study as individual, academic, initial licensure, and environmental factors that may influence NCLEX-RN outcomes. Although the model was originally intended for USENs, it has a universal applicability [10].

### 1.2. NCLEX-RN and the IEN

Research on the NCLEX-RN and PENs is very limited. In a scoping review of 17 studies on factors affecting IEN NCLEX-RN performance published from 1994 to 2020, the use of the English language, differences in nursing education internationally, and level of NCLEX-RN familiarity were identified as common factors affecting IEN NCLEX-RN performance [8,11]. Nursing work experience, support system, and the Commission on Graduates of Foreign Nursing Schools certification examination (CGFNS CE) were found to predict IENs passing the NCLEX-RN on their first attempt [8,11].

In April 2023, 25% of Next Generation NCLEX-RN used new format questions that simulate actual clinical scenarios and measured clinical judgment [12]. The NCLEX-RN blueprint is revised every three years to reflect current U.S. nursing practice [13]. As patient care becomes more complex, the NCLEX-RN reflects an increased threshold for passing, making it more difficult to pass [14]. The NCLEX-RN passing thresholds were increased in 2008, 2012, and 2016. This change resulted in a drop in NCLEX-RN pass rates. In 2012, the national first-time pass rate was 90.3% and dropped to 84.6% in 2016 [14].

Differences in nursing education and nursing practice internationally have limited IENs’ familiarization of the NCLEX-RN questions and impacted their test outcomes [9,15,16]. Characteristics unique to PENs may influence NCLEX-RN preparation and outcomes. U.S. regulatory bodies require additional documents and fees (i.e., high school diploma, English proficiency exams, credentials evaluation, and international testing fee) when IENs apply for the NCLEX-RN. Thus, they invest significant time and resources to prepare for the NCLEX-RN, which is described as stressful and affects their NCLEX-RN preparation and outcomes. Failing the NCLEX-RN has significant individual, family, and socioeconomic implications [9]. Additionally, compared to USEN, PENs have to have taken a nurse licensure exam (NLE), earned a graduate nursing degree, and worked as registered nurses domestically or internationally before taking the NCLEX-RN. However, the association of these variables on PENs passing the NCLEX-RN on their first attempt has not been studied.

#### 1.2.1. Contextual Characteristics

Previous studies reported that age, gender, race, use of the English language, and family/work responsibilities affect NCLEX-RN outcomes [15,17,18,19,20]. NCLEX-RN candidates who are African American, Asian, Hispanic, and African, younger [17], and those with family/work responsibilities [18] during the test preparatory stage had lower pass rates. IENs from countries with English as the official language had higher NCLEX-RN FTPR, compared to those from countries with official languages other than English [20]. IENs with a support system during their preparation for the licensure exam had higher chances of passing the exam [18,21].

#### 1.2.2. Academic Factors

A baccalaureate degree in nursing is the only educational entry pathway to practice nursing in the Philippines. Although there is a higher percentage of IENs with a bachelor’s degree in nursing compared to USENs [5,22], this does not lead to having a higher NCLEX-RN FTPR. Variations in academic preparation and testing are challenging to PENs due to a lack of NCLEX-RN familiarity and difficulty with prioritization, delegation, and pharmacology [9]. This variation is related to practice differences in the U.S. and internationally [9]. In some countries, nursing curricula are outdated and use the traditional medical model [23,24] and nursing practice is mainly dependent on physicians, which is entirely different from the autonomy, management, and clinical judgment skills that USENs learn and practice. This can be a hindrance in answering management-of-care questions, which account for 17–23% of NCLEX-RN questions [13].

Limited clinical experience in nursing schools was detrimental to PEN NCLEX-RN performance [9]. While some PENs possess a graduate nursing degree, this did not provide enough confidence when taking the NCLEX-RN, especially when their focus of specialization, such as nursing administration, primary health care, or community health, was not focused on clinical nursing courses that are critical areas in the exam [5,9,25]. Furthermore, students who chose nursing as their first career choice tend to be more successful academically compared to those who chose a different program [26].

In U.S. studies, public nursing schools and those with a higher percentage of full-time faculty members have higher NCLEX-RN FTPR [27]. Research conducted in the Philippines found that graduates of public institutions had higher PNLE FTPR compared to graduates of private nursing institutions [28,29].

Moreover, due to differences in nursing education internationally, additional requirements for IEN NCLEX-RN application are needed, such as secondary school information, CGFNS CE or credentials evaluation, and English proficiency exams [30]. PENs described the time, process, and cost of meeting these requirements as challenges when preparing for the NCLEX-RN, which may have impacted test outcomes [9].

#### 1.2.3. Initial Nursing Licensure Factors

Initial nursing licensure in this context refers to their first time seeking licensure in their country of nursing education prior to taking the NCLEX-RN. PENs claimed that PNLE preparation was helpful to their NCLEX-RN preparatory experiences and outcomes [9]. However, NCLEX-RN familiarization programs and readiness assessments are lacking in Philippine nursing schools, but they have partnerships with review centers that focus on PNLE preparation [31]. Thus, PENs preparing for NCLEX-RN seek review centers or online resources independently. Although the Philippines has NLE, the exams are completely different compared to NCLEX-RN [24,32]. There is a lack of published research on the association of PNLE and NCLEX-RN passing on the first attempt.

#### 1.2.4. Employment Factors

Most IENs have professional nursing experience before taking the NCLEX-RN. However, differences in international nursing practice may create confusion when studying for the NCLEX-RN and may have accounted for a drop in Canadian FTPR, from 87% to 69.7%, when the NCLEX-RN was first adopted in Canada in 2015 [33,34,35]. The length and nature of nursing employment have an impact on NCLEX-RN outcomes. In a Canadian study, IENs with three to five years of nursing experience before taking the NCLEX-RN have higher odds of passing the NCLEX-RN [21]. Nursing workplace influenced positive PEN NCLEX-RN preparatory experiences and outcomes [9].

In summary, the available literature on international NCLEX-RN studies was conducted mostly on IENs outside the Philippines. There is very limited research investigating the association of individual, academic, initial nursing licensure, and employment factors on NCLEX-RN passing of the largest population comprising international NCLEX-RN applications. Furthermore, there is a need to analyze the contextual characteristics of PENs to promote a deeper understanding of factors associated with their NCLEX-RN performance.

## 2. Materials and Methods

### 2.1. Research Design

A retrospective correlational research design was used in this study. This design is appropriate for determining relationships between variables [36]. In this study, individual, academic, initial nursing licensure, and employment factors were the independent variables, while NCLEX-RN performance, categorized as pass or fail, was the dependent variable. The consensus-based Checklist for Reporting of Survey Studies (CROSS) [37] was used to guide the reporting of research findings.

### 2.2. Sample and Sampling

G*Power analysis required 128 participants to yield a power of 0.80, a two-tailed test of 0.05 level of significance, and an effect size of 0.30. PENs were selected for this study since almost 60% of IEN NCLEX-RN applications come from the Philippines [13]. Participants were recruited online through professional groups on social media (Facebook), such as Registered Nurses in the Philippines and Lefora Filipino Nurses to the U.S., and professional organizations, such as the Philippines Nurses Association of America. Inclusion criteria include an age of at least 18 years, graduate of bachelor’s degree in nursing in the Philippines, and having taken the computer-based NCLEX-RN. Through single-stage convenience sampling, 328 participants started the survey. Follow-up on incomplete surveys was carried out one week after the initial survey. A total of 159 participants completed the survey. The email addresses of completed surveys were checked to avoid duplication of responses. Data from 19 participants that were missing at random—not due to systematic factors—were not included in the study. A total of 146 participants were included in this study (44.5% completion rate).

### 2.3. Data Collection

Due to limited studies in this area and the unavailability of instruments to measure the research variables, a literature-based survey questionnaire was developed. Twenty-three questions regarding individual (three questions), academic (six questions), initial nursing licensure (ten questions), and employment factors (four questions) were asked. Two PhD-prepared nurse educators who are familiar with Philippine nursing education and the NCLEX-RN assessed the content validity of the survey questionnaire. The instrument was not assessed for reliability since the variables are factual items (i.e., demographic information, NCLEX-RN pass/fail status, etc.), not a multi-item psychological scale. The web-based survey was administered online via Qualtrics on professional nursing groups on several social media platforms.

### 2.4. Data Analysis

Data analysis was performed on R4.2 software. Descriptive statistics such as frequency, percentage distribution, mean, and standard deviation described the NCLEX-RN results and the individual, academic, initial nursing licensure, and employment factors of PENs who passed or failed the NCLEX-RN on the first attempt. The Chi-squared test (or Fisher’s exact test when the cell count was less than six) was used to determine the relationship between the categorical independent and dependent variables. All statistical tests were computed at a 0.05 level of significance. Missing data occurred in age, nursing degree, and employment variables. The missing data were modeled as an independent category in the statistical analyses.

### 2.5. Ethical Considerations

The Institutional Review Board of the University of North Carolina at Charlotte, under the Office of Research Protections and Integrity, approved this study (IRB 19-108).

## 3. Results

### 3.1. Participant Characteristics

The majority of the 146 participants were female (70.5%, *n* = 103) and single (56.8%, *n* = 83), and their mean age was 28.2 years (*SD* = 5.68) (Table 1). Most participants graduated from a private school (87%, *n* = 127) and do not have a graduate nursing degree (83.6%, *n* = 122). Nursing was the first career choice of most participants (95.9%, *n* = 140). Most participants passed the PNLE on their first attempt (95.9%, *n* = 140) and did not take the CGFNS-CE (69.2%, *n* = 101). The majority self-reviewed for the NCLEX-RN (54.8%, *n* = 80) and almost half (45.2%, *n* = 66) spent less than three months reviewing for the NCLEX-RN. The majority of the participants were employed (77.4%, *n* = 113), had a full-time job (68.5%, *n* = 100), and worked in the field of nursing (64.4%, *n* = 94) and in a hospital setting (46.6%, *n* = 68) when they took the NCLEX-RN.

### 3.2. Association of Demographic, Academic, Initial Nursing Licensure, Employment Factors, and Passing NCLEX-RN on First Attempt

The majority of participants (79.5%, *n* = 116) passed the NCLEX-RN on their first attempt. All demographic, academic, initial nursing licensure, and employment factors, except for PNLE attempts and workplace, were not significantly associated with passing the NCLEX-RN on the first attempt (Table 2). The demographic variables of gender [χ^2^ (1, *n* = 146) = 1.10, *p* = 0.293] and marital status [χ^2^ (1, *n* = 146) = 1.12, *p* = 0.291] were not significantly associated with PENs passing the NCLEX-RN on their first attempt. Academic factors such as the type of school ownership [Fisher’s, *p* = 0.365], nursing degree [Fisher’s *p* = 0.083], and nursing as a first career choice [Fisher’s *p* = 0.101] were not significantly associated with PEN NCLEX-RN passing on first attempt.

Passing the PNLE the first time is significantly associated with passing the NCLEX-RN on the first attempt [Fisher’s *p* = 0.001]. Initial nursing licensure factors such as taking the CGFNS certification exam [χ^2^ (1, *n* = 146) = 0.11, *p* = 0.741] and the type [χ^2^ (1, *n* = 146) = 0.72, *p* = 0.396] and duration [χ^2^ (2, *n* = 146) = 3.21, *p* = 0.201] of NCLEX-RN preparation were not significantly associated with PENs passing the NCLEX-RN on the first attempt. Employment factors such as employment status [Fisher’s *p* = 0.196], type of employment [χ^2^ (2, *n* = 146) = 0.09, *p* = 0.958], and field of employment [χ^2^ (2, *n* = 146) = 2.17, *p* = 0.337] were not significantly associated. However, workplace [Fisher’s *p* = 0.026] was significantly associated with PEN NCLEX-RN performance on the first attempt. Participants who worked in non-hospital settings (93.8%, *n* = 30) have higher NCLEX-RN pass rates compared to those who worked in hospitals (79.4%, *n* = 54).

Overall, except for NLE attempt and workplace during the test, all individual, academic, and employment factors were not significantly different between PENs who passed or failed the NCLEX-RN on first attempt.

## 4. Discussion

This study aimed to describe the individual, academic, initial nursing licensure, and employment factors of PENs who passed or failed the NCLEX-RN on their first attempt and to determine the association between the participants’ contextual characteristics and passing the NCLEX-RN on the first attempt.

The majority of participants passed the NCLEX-RN on their first attempt. Results show that individual characteristics (gender and marital status), academic factors (nursing as first career choice, type of nursing school, and graduate nursing degree), initial licensure factors (taking the CGFNS-CE and NCLEX-RN review preparation), and employment factors (type and status of employment) were not significantly associated with passing the NCLEX-RN on the first attempt among PENs. In this study, passing the PNLE on the first attempt and workplace (i.e., employment in non-hospital settings) were the only independent variables that were significantly related to PENs passing the NCLEX-RN on their first attempt. The results of this study provide preliminary information on this understudied but relevant research area that has implications for Philippine nursing education and regulation, being the primary source of IEN in the U.S. nursing workforce.

### 4.1. Contextual Factors

A higher percentage of PENs who passed the NCLEX-RN on their first attempt were male, single, and had nursing as their first career choice. However, the association between these factors and passing the NCLEX-RN is not statistically significant. The findings of this study regarding the influence of gender on the NCLEX-RN are contrary to most studies. This may be related to methodological and contextual differences. The proportion of the male-to-female nursing student population internationally is higher compared to the U.S. nursing student population [38,39]. While males comprised 30.7% of the participants in this study, published NCLEX-RN studies acknowledged the underrepresentation of the male gender in their samples as a study limitation [40].

Results showed that a higher percentage of single NCLEX-RN candidates passed the NCLEX-RN on their first attempt compared to married candidates. Being married may pose numerous challenges related to family roles and responsibilities when preparing for the NCLEX-RN. Married individuals may have less time to study due to household and childcare responsibilities. In the cultural context of the participants, the family provider role is a major responsibility and expectation. This finding supports previous studies of IENs from Korea, Mexico, and the Philippines on the influence of family responsibilities on NCLEX-RN outcomes [9,18,20].

Those who had nursing as a first career choice had a higher NCLEX-RN pass rate compared to those who did not choose nursing as their first career option. This choice serves as a motivation to perform better in their nursing education and career. This finding is related to Salamonson’s study, which claimed that nursing as a first career choice is associated with nursing program success [26].

### 4.2. Academic Factors

The results show that a greater percentage of NCLEX-RN candidates who graduated in nursing from public schools passed the NCLEX-RN on their first attempt compared to those who completed nursing education in private institutions. The influence of institutional characteristics and nurse licensure outcomes is reported in previous U.S. [27] and Philippine studies [28,29], which claimed that nursing graduates from public nursing institutions had higher first-time pass rates in the NCLEX-RN and NLE, respectively. However, no significant association was found between the type of institution and NCLEX-RN outcomes in our study. The small sample size of graduates of public schools may be a limitation in establishing this association.

A graduate nursing degree provides advanced nursing knowledge and skills that are useful in answering complex NCLEX-RN questions. Although more PEN NCLEX-RN candidates with graduate nursing degrees passed the NCLEX-RN on their first attempt, graduate nursing education and passing the NCLEX-RN were not significantly related. This can be attributed to the small sample size of participants with graduate degree who failed the NCLEX-RN.

### 4.3. Initial Licensure Factors

Most PENs who passed the NCLEX-RN the first time had passed the PNLE on their first attempt. This is the first study that established a statistically significant association between passing the PNLE and NCLEX-RN on the first attempt. Although a higher percentage of NCLEX-RN pass results was observed in PENs who enrolled in review centers and studied for four to six months, these preparations were not significantly associated with passing the NCLEX-RN on the first attempt.

The experience of preparing for and passing the PNLE is a prelude to international nursing examinations. For nursing programs internationally that are patterned after the U.S. nursing curriculum, such as the Philippines [41], successfully passing the PNLE may contribute to successful NCLEX-RN performance [9]. The Philippines has nursing review centers that prepare their graduates for the PNLE [31]. Those centers offering NCLEX-RN review programs present a viable option for NCLEX-RN preparation. This finding supports a study on Korean IENs [18], which reported that NCLEX-RN preparatory classes were helpful in passing the NCLEX-RN.

Most PENs who passed the NCLEX-RN on their first attempt studied for four to six months. The differences in nursing education, nursing practice, and unfamiliarity with the NCLEX-RN create a need for PENs to have a longer preparation time for the examination. While most USENs take the NCLEX-RN one to two months after graduation, it is different for PENs. Aside from learning nursing practice in the U.S., PENs need to learn the NCLEX-RN test plan, test questions, and the use of computers for testing, which need a longer NCLEX-RN preparatory time.

### 4.4. Employment Factors

There was a higher percentage of NCLEX-RN passes on the first attempt for PENs who were not employed at the time of examination. Since preparing for the NCLEX-RN is an expensive investment, some PENs may prioritize preparing for the NCLEX-RN to avoid wasting time and financial resources; thus, they opt to either delay getting employed or resign from their jobs while preparing for the NCLEX-RN [9]. Having a full-time job and working for long hours while preparing for the NCLEX-RN reduces time to study, which can have negative effects on academic performance and test outcomes [42].

For those who were employed at the time of NCLEX, a higher percentage of passing was seen among those who were worked full-time and employed in nursing. Working as staff nurses provides actual patient care experiences that enhance clinical nursing knowledge and skills that help in developing critical thinking while preparing for NCLEX-RN [9,21]. Active involvement in actual clinical situations helps foster clinical judgment, which is critical in analyzing NCLEX-RN questions [13]. The workplace was significantly associated with passing the NCLEX-RN. Interestingly, PENs who were employed in non-hospital settings, such as schools of nursing, at the time of NCLEX-RN had higher first-time pass rates. Intuitively, one who teaches as a faculty member in a nursing school has more cognitive preparation and skills to pass the NCLEX-RN.

To our knowledge, this is the first study that describes the association between PENs’ contextual, academic, initial nursing licensure, and nursing employment factors, and passing the NCLEX-RN on the first attempt. The results of this study contribute to the body of knowledge that helps form an understanding of the variability of factors unique to the PEN population.

### 4.5. Implications and Recommendations

#### 4.5.1. PEN NCLEX-RN Candidates

Recognizing individual characteristics that are associated with PENs passing the NCLEX-RN on the first attempt is important because of the cost and time needed for test preparation and application. IENs have more requirements and fees when applying for the test compared to USENs. For example, some U.S. boards of nursing require CGFNS CE and English proficiency exams before taking the NCLEX-RN. The processing and cost of additional IEN-specific required documents and international testing fees (USD 200) are needed for IENs’ NCLEX-RN applications. Travel outside their home country may be required when scheduling the NCLEX-RN due to limited schedules in international testing centers. Furthermore, within the context of nurses in the Philippines, passing the NCLEX-RN on the first attempt promotes socioeconomic mobility and professional recognition. Thus, passing the NCLEX-RN on the first attempt is cost-effective and rewarding.

With the availability of standardized assessment tests, online materials, and support groups that are essential to NCLEX-RN success, PENs may consider adapting these resources to assess NCLEX-RN readiness, improve test outcomes, and promote an overall positive NCLEX-RN preparatory experience [43,44].

#### 4.5.2. Nursing Policy and Regulation

Without an international benchmark, the NCLEX-RN has served as a measure to compare nursing education internationally. While most countries do not prepare their graduates for U.S. nursing practice, some countries, such as the Philippines, intentionally design nursing programs to train their students for the global labor market. The representation of 187 countries and territories on the NCLEX-RN is evidence of the interest of nurses globally to work in the U.S. For IEN source countries, this study’s findings provide focus points for designing educational supports and regulatory policies aimed at increasing IENs’ success in passing the NCLEX-RN. In the Philippine context, the Commission and Higher Education’s Technical Panel for Nursing Education and the Board of Nursing may recommend curricular and programmatic changes to achieve this goal.

Since nursing education and nursing practice are not well regulated in other countries (i.e., lack of national nursing law, nursing regulation, or NLE as a requirement to practice nursing), it is crucial for nursing regulatory bodies to design policies that will help elevate the standards of nursing education, practice, and regulation in their respective countries [45]. Some countries have gaps and deficiencies in their nursing curriculum, while other countries allow nursing graduates to work right after graduation, without taking a national licensure examination. Regulating nursing practice internationally may improve individual IEN, nursing school, and country NCLEX-RN pass rates.

Other than the test itself, the NCLEX-RN requirements are resource-intensive and the application and processing time are tedious and stressful. The long processing time has created a longer time lag for IENs to take the NCLEX-RN. These barriers negatively affect IEN NCLEX-RN preparation and outcomes. Reducing or eliminating some of these barriers, without compromising public safety, may be more likely to produce a more positive NCLEX-RN preparatory experience and outcome. With the cyclical U.S. nursing shortage and the COVID-19 pandemic, there is a need for U.S. boards of nursing to streamline NCLEX-RN application requirements and expedite processing times.

#### 4.5.3. Nursing Education, Practice, and Research

Since passing the PNLE on the first attempt is associated with NCLEX-RN first-time passing, academic preparation in nursing school should be given appropriate attention. With more than half of PENs failing the NCLEX-RN on their first attempt, understanding academic factors that influence PENs passing the NCLEX-RN is critical in designing educational interventions to increase the chances of passing the exam. As the main source of international NCLEX-RN applications, undergraduate nursing programs in the Philippines should provide opportunities to familiarize nursing students with the NCLEX-RN test blueprint, type of questions, and computer-adaptive testing, since familiarity with the NCLEX-RN is essential in passing the exam. Moreover, there is a need to introduce Next Generation NCLEX (NGN) to nursing students because NGN started in April 2023. Furthermore, as NGN focuses on clinical judgment, nursing schools should emphasize the use of active learning strategies such as simulations, case studies, reflection, and clinical practice to develop clinical judgment, which is critical in passing the NCLEX-RN [45].

The unique context of PENs who are professional nurses at the time of the NCLEX-RN may be considered as both a facilitating and hindering factor. As a facilitating factor, nursing employment offers exposure to clinical scenarios that may help in developing clinical judgment, which is essential in answering NCLEX-RN questions. As a hindrance, nursing employment may reduce test preparatory time. This may lead to reduced nursing working hours or resignation from nursing positions to prepare for the NCLEX-RN, potentially impacting nursing staffing.

Further studies to include other IENs with a larger sample size are needed. Future researchers need to explore the influence of other factors affecting IEN NCLEX-RN performance. Variables such as academic performance, course grades, licensure exam scores, and critical thinking have not been studied for PENs. Moreover, the adaptation of the NURS model is needed to develop an IEN-specific conceptual model to better understand the NCLEX-RN from an IEN perspective.

### 4.6. Study Limitations

The limitations of this study include its focus only on PENs, convenience sampling, representativeness of the sample, and instrumentation. Since the study specifically studied PENs, the findings cannot be generalized to the IEN population. Convenience sampling may have limited the representativeness of the sample. In this study, PENs who passed the NCLEX-RN the first time were overrepresented, constituting a sample bias. Future research should involve IENs from other countries and an investigation of IENs who failed the NCLEX-RN on the first attempt. Due to the different contextual characteristics of IENs, future studies should include the development of valid and reliable IEN-specific research instruments.

## 5. Conclusions

This study aimed to determine the association between academic, initial nursing licensure, and employment factors, and PENs passing the NCLEX-RN on the first attempt. The limited research on this topic and the low NCLEX-RN pass rates of PENs were the main impetus for conducting this research as an attempt to build a conceptual base on this understudied area. PENs have unique characteristics that relate to their NCLEX-RN outcomes. Passing the national licensure examination of origin on the first attempt and employment in non-hospital settings showed significant associations with passing the NCLEX-RN on the first attempt. Emphasis should be placed on the academic preparation of undergraduate nursing students in order to assist them in passing the national licensure examination on their first attempt. Careful selection of clinical areas for practice is essential in having adequate and appropriate opportunities to enhance clinical judgment.

Awareness of the association of these factors and NCLEX-RN performance provides direct benefit to individual PENs and has relevant implications to faculty members of undergraduate and graduate nursing programs, the administration of health care facilities, and the nursing workforce in general.

## Figures and Tables

**Table 1:** Participant characteristics.

Demographic Characteristics	*n* (%)/Mean (SD) *
**Demographic**	
**Gender**	
Male	43 (29.5)
Female	103 (70.5)
**Marital Status**	
Single	83 (56.8)
Married	63 (43.2)
**Age** (range: 19–53)	28.2 (5.68) *
**Academic**	
**Type of ownership**	
Public school	19 (13.0)
Private school	127 (87.0)
**Nursing degree**	
Undergraduate	122 (83.6)
Graduate	21 (14.4)
**Nursing as first career choice**	
Not first	6 (4.1)
First	140 (95.9)
**Initial licensure**	
**PNLE attempts**	
Once	140 (95.9)
More than once	6 (4.1)
**CGFNS before NCLEX**	
Not taken	101 (69.2)
Taken	45 (30.8)
**Type of NCLEX preparation**	
Assisted	66 (45.2)
Self-review	80 (54.8)
**Duration**	
1–3 months	68 (46.6)
4–6 months	47 (32.2)
Over 6 months	31 (21.2)
**Employment**	
**Status**	
Unemployed	32 (21.9)
Employed	113 (77.4)
**Type**	
Part-time or less	42 (28.8)
Full-time	100 (68.5)
**Field**	
Non-nursing	47 (32.2)
Nursing	94 (64.4)
**Workplace**	
Other	32 (21.9)
Hospital	68 (46.6)

Note: PNLE—Philippine Nurse Licensure Exam; CGFNS—Commission on Graduates of Foreign Nursing Schools; NCLEX—National Council Licensure Examination–Registered Nurses; * SD–Standard deviation

**Table 2:** Association of demographic, academic, initial nursing licensure, employment factors, and passing NCLEX-RN on first attempt.

	NCLEX-RN First Attempt (*n* = 146)		
	Pass (*n* = 116) *n* (%)	Fail (*n* = 30) *n* (%)	Chi-Squared (χ^2^) or Fisher’s Exact Test (*p*-Value)
**Demographic**			
**Gender**			χ^2^ = (1, 146) = 1.10, *p* = 0.293
Male	37 (86.0)	6 (14.0)	
Female	79 (76.7)	24 (23.3)	
**Marital Status**			χ^2^ (1, 146) = 1.12, *p* = 0.291
Single	69 (83.1)	14 (16.9)	
Married	47 (74.6)	16 (25.4)	
**Age** (range: 19–53)	27.9 (5.43)	29.4 (6.52)	0.187
**Academic**			
**Type of ownership**			Fisher’s *p* = 0.365
Public school	17 (89.5)	2 (10.5)	
Private school	99 (78.0)	28 (22.0)	
**Nursing degree**			Fisher’s *p* = 0.083
Undergraduate	94 (77.0)	28 (23.0)	
Graduate	20 (95.2)	1 (4.8)	
**Nursing choice**			Fisher’s *p =* 0.101
Not first	3 (50.0)	3 (50.0)	
First	113 (80.7)	27 (19.3)	
**Initial licensure**			
**PNLE attempts**			Fisher’s *p* = 0.001 *
Once	111 (82.8)	23 (17.2)	
More than once	1 (16.7)	5 (83.3)	
**CGFNS before NCLEX**			χ^2^ (1, 146) = 0.11, *p* = 0.741
Not taken	79 (78.2)	22 (21.8)	
Taken	37 (82.2)	8 (17.8)	
**Type of preparation**			χ^2^ (1, 146) = 0.72, *p* = 0.396
Assisted	55 (83.3)	11 (16.7)	
Self-review	61 (76.2)	19 (23.8)	
**Duration**			χ^2^ (2, 146) = 3.21, *p* = 0.201
1–3 months	53 (77.9)	15 (22.1)	
4–6 months	41 (87.2)	6 (12.8)	
Over 6 months	22 (71.0)	9 (29.0)	
**Employment**			
**Status**			Fisher’s *p* = 0.196
Unemployed	27 (84.4)	5 (15.6)	
Employed	89 (78.8)	24 (21.2)	
**Type**			χ^2^ (2, 146) = 0.09, *p* = 0.958
Part-time or less	33 (78.6)	9 (21.4)	
Full-time	80 (80.0)	20 (20.0)	
**Field**			χ^2^ (2, 146) = 3.21, *p* = 0.337
Non-nursing	34 (72.3)	13 (27.7)	
Nursing	78 (83.0)	16 (17.0)	
**Workplace**			Fisher’s *p =* 0.026 *
Other	30 (93.8)	2 (6.2)	
Hospital	54 (79.4)	14 (20.6)	

Note: * Statistically significant at *p* = 0.05; PNLE—Philippine Nurse Licensure Exam; CGFNS—Commission on Graduates of Foreign Nursing Schools; NCLEX—National Council Licensure Examination–Registered Nurses.

## Data Availability

Data can be directly requested from the corresponding author.

## Abbreviations

The following abbreviations are used in this manuscript:

IEN	Internationally educated nurses
NCLEX-RN	National Council Licensure Examination—Registered Nurses
NLE	Nurse licensure examination
PEN	Philippine-educated nurses
